# Alternative Polyadenylation: Methods, Findings, and Impacts

**DOI:** 10.1016/j.gpb.2017.06.001

**Published:** 2017-10-12

**Authors:** Wei Chen, Qi Jia, Yifan Song, Haihui Fu, Gang Wei, Ting Ni

**Affiliations:** State Key Laboratory of Genetic Engineering & MOE Key Laboratory of Contemporary Anthropology, Collaborative Innovation Center of Genetics and Development, School of Life Sciences and Shanghai Cancer Center, Fudan University, Shanghai 200438, China

**Keywords:** Alternative polyadenylation, Next-generation sequencing, 3′UTR, Alternative splicing, Gene regulation

## Abstract

**Alternative polyadenylation** (APA), a phenomenon that RNA molecules with different 3′ ends originate from distinct polyadenylation sites of a single gene, is emerging as a mechanism widely used to regulate gene expression. In the present review, we first summarized various methods prevalently adopted in APA study, mainly focused on the **next-generation sequencing** (NGS)-based techniques specially designed for APA identification, the related bioinformatics methods, and the strategies for APA study in single cells. Then we summarized the main findings and advances so far based on these methods, including the preferences of alternative polyA (pA) site, the biological processes involved, and the corresponding consequences. We especially categorized the APA changes discovered so far and discussed their potential functions under given conditions, along with the possible underlying molecular mechanisms. With more in-depth studies on extensive samples, more signatures and functions of APA will be revealed, and its diverse roles will gradually heave in sight.

## Introduction

Polyadenylation, the cleavage of 3′ end of precursor mRNA (pre-mRNA) and the sequential addition of a poly(A) tail wherein, is the last key step in mRNA maturation process, which is important for the translation efficiency, stability, and localization of mRNA [1], [2], [3]. Almost all eukaryotic mRNA and many non-coding RNAs (ncRNAs) are polyadenylated. Many eukaryotic genes contain more than one polyA (pA) sites, termed as alternative polyadenylation (APA), leading to the generation of distinct mRNA isoforms from the same gene [4], [5]. According to the location of pA sites along genes, APA can be classified into two major categories. In the first scenario, alternative pA sites are located in internal exons or introns, which affects coding regions and leads to the generation of protein isoforms with distinct C termini, thus called coding region-APA (CR-APA) [6]. In the other scenario, alternative pA sites are located in the 3′ untranslated region (3′UTR), which leads to the generation of transcripts with the same coding frame but variable 3′UTRs, thus called UTR-APA. It should be noted that, although the UTR-APA isoforms do not change the coding frame, it might lead to changes in mRNA half-life, translation efficiency, *etc.*, since the longer 3′UTR can have more microRNA (miRNA)-binding sites, more RNA-binding protein (RBP) recognition sites, or altered RNA secondary structure [2], [3], [7], [8], [9].

Genes encoding immunoglobulin M (*IgM*) and dihydrofolate reductase (*DHFR*) are among the earliest genes that were discovered to have APA in 1980 [10], [11]. Since then, evidence for the presence of APA had been accumulating slowly in literature, and about 95 genes had been reported with APA by 1997 [12]. With the increasing number of expressed sequences in public databases, global profiling of APA has been performed through bioinformatics analyses of the expressed sequence tags (ESTs) [4], [13]. Global APA changes have also been detected through microarray-based approaches [14], [15], [16], [17] and paired-end ditag (PET) analyses [18], [19]. With the progress of next-generation sequencing (NGS) technology and the rapid accumulation in expression sequence data, several bioinformatics methods have been developed to identify APA events in RNA-seq data [20], [21], [22], [23]. However, only reads mapped to the 3′ ends of the mRNA are useful for APA discovery [22], leading to insufficient coverage on the regions of interest. Thus NGS-based techniques special for pA site identification have been developed using different strategies [2]. Ever since, many genes have been reported to possess multiple pA sites in many species [2]. APA has been demonstrated to be one of the major contributors to transcriptome and proteome diversity, and play important roles in many biological processes [24], [25].

Given the widespread usage and great importance of NGS-based methods for APA identification, in the present review, we first summarized most of these RNA-seq methods specifically adapted for APA analysis up to date, the bioinformatic analysis approaches adopted in subsequent analysis, and strategies suitable for APA study at the single-cell level. We then reviewed the rapid progress in APA studies in many fields mainly based on such methods, categorized the APA events, and discussed their potential functions and the possible mechanisms in APA regulation. In addition, some promising/interesting foresights are proposed according to the new advances in APA studies. Compared to previous APA review articles [2], [3], [6], [8], [9], this review focuses on the deep sequencing-based methods and the latest progress in the field of APA.

## NGS-based methods for APA identification

RNA-seq has been used as the routine method for transcriptome profiling in the past decade, due to its advantages in detection of new genes, much wider dynamic detection ranges for gene expression quantification, and single-base resolution [24], [26], [27]. Consequently, large numbers of unannotated genes and non-coding RNA (ncRNAs) with alternative splicing (AS) patterns have come to the fore [24]. However, because of the relatively low overall read coverage of 5′ and 3′ ends of genes, RNA-seq is not suitable for identifying pA sites precisely and extensively. Thus, many library construction methods have been developed to enrich fragments carrying poly(A) tail, followed by high-throughput sequencing, which are generally known as 3′-enriched RNA-seq [7], [28], [29], [30], [31], [32], [33], [34], [35], [36], [37], [38], [39], [40], [41], [42], [43], [44], [45], [46], [47], [48]. These methods outperform standard RNA-seq in pA site identification and show advantages in quantifying isoforms with 3′UTRs of different lengths. As to now, 3′-enriched RNA-seq protocols can be classified mainly into two categories based on the strategy used to enrich the 3′-termini of transcripts, that is, oligo(dT) priming-based methods [7], [30], [31], [32], [33], [35], [36], [37], [38], [39], [40], [41], [42], [44], [45], [46], [47], [48] and RNA manipulation-based methods [29], [43].

**Figure 1:**
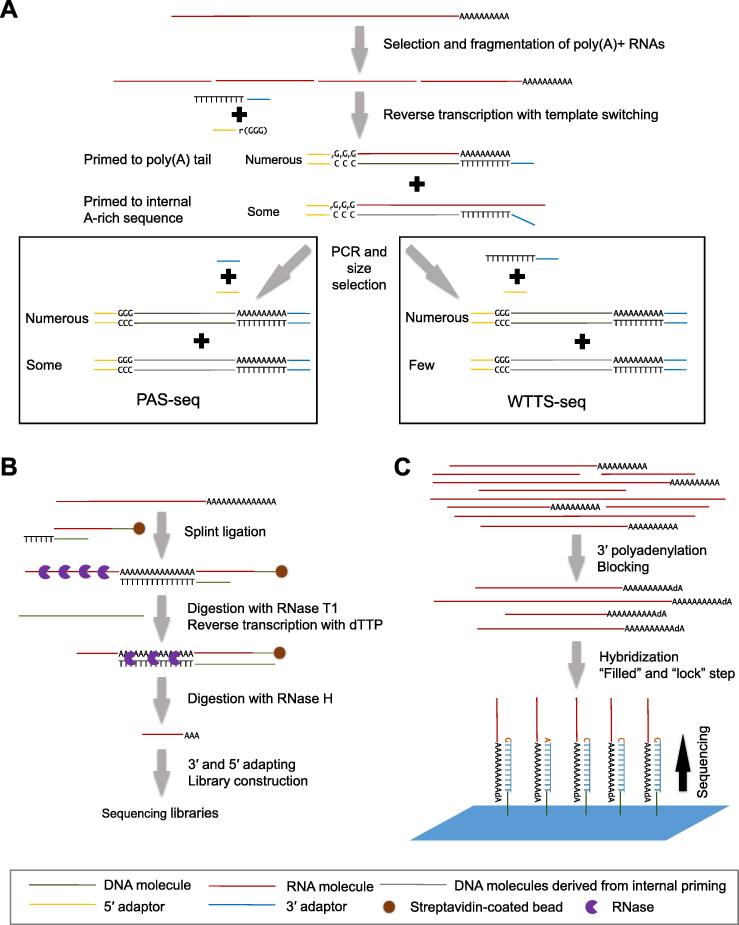
**Schematic diagram illustrating the library preparation procedure using three major categories of 3′-enriched RNA-seq methods** **A.** Schematic of two representative oligo(dT) priming-based methods, PAS-seq (on the left) and WTTS-seq (on the right). Poly(A)+ RNAs are selected and fragmented, and the resulting fragments of 3′-termini are enriched by oligo(dT) primer and converted to cDNA via reverse transcription. After size selection, PCR is performed with universal primer for PAS-seq (left); alternatively, PCR could be carried out with oligo(dT)-containing primers to alleviate internal priming for WTTS-seq (right). **B.** Schematic of 3P-seq, an RNA manipulation-based method. Poly(A)+ RNAs are first ligated to a splint adaptor with the single-stranded part of RNA molecules subjected to digestion with RNase T1; the resulting fragments of 3′-termini are purified with streptavidin-coated beads and reverse transcription is then carried out with dTTP. The RNA–DNA duplex is digested with RNase H. After purification, RNA fragments containing pA site are ligated with 3′ and 5′ adaptors followed by the traditional NGS library construction procedures. **C.** DRS procedure. Poly(A)+ RNA molecules are blocked and hybridized with oligo(dT) primer fixed at the sequencing surface. Afterward, poly(A) tails of RNA molecules are filled with dTTP (in blue), and a locking step is conducted to stop subsequent nucleotide addition (in brown). This figure is redrawn based on the principles of published protocols [28], [29], [33], [42]. Purple 3/4 circles, RNase T1 or RNase H; Blue patch, oligo(dT) coated slide. PAS-seq, poly(A) site sequencing; WTTS-seq, whole transcriptome termini site sequencing; 3P-seq, poly(A)-position profiling; DRS, direct RNA sequencing.

Besides 3′-enriched RNA-seq, some global APA studies have also been carried out via direct RNA sequencing (DRS) [26], [32], [47] (Figure 1). DRS is a third-generation sequencing method that can only be adapted for Helicos single molecule sequencing platform. Given DRS is no longer available due to the bankrupt of the instrument provider, we thus discuss the remaining two categories of 3′-enriched RNA-seq methods in the following context. Details of some representative methods in each category and an overall comparison are summarized in Table 1.

**Table 1:** **Advantages and disadvantages of methods applied to detect pA sites**

**Category**	**Methods**	**Advantages**	**Disadvantages**	**Refs.**
Oligo(dT) priming-based	Mangone et al.; Wu et al.; PAS-seq; poly(A)-seq; PA-seq; 3′ seq; A seq; 3PC; 3′T fill; 3SEQ; EXPRSS; MAPS SAPAS; WTTS-seq	Easy to implement; Time-saving; Preserving strand information; Able to handle hundreds of samples simultaneously	Internal priming;	[31], [32], [33], [35], [36], [37], [39], [40], [41], [42], [46], [47], [49], [50]
			Low diversity sequencing libraries; Bias-prone; Loss of small fragments during size selection	

RNA manipulation-based	3P-seq; 3′ READS; PAT-seq	Bypassing internal priming; Revealing authentic pA sites; Preserving strand information	Time consuming; Laborious; Involving multiple steps of RNA manipulation; Bias-prone	[29], [30], [43]


*Note*: PAS-seq, poly(A) site sequencing; PA-seq, polyadenylation sequencing; 3PC, 3′ poly(A) site mapping using cDNA circularization; EXPRSS, expression profiling through randomly sheared cDNA tag sequencing; MAPS, multiplex analysis of polyA-linked sequences; SAPAS, sequencing APA sites; WTTS-seq, whole transcriptome termini site sequencing; 3P-seq, poly(A)-position profiling; 3′ READS, 3′ region extraction and deep sequencing; PAT-seq, poly(A)-test RNA-sequencing.

### Oligo(dT) priming-based methods

Most methods in this category adopt the polyadenylation feature (polyA tail) of mRNA as a hook to capture their 3′-termini. Typically, mRNA molecules are reversely transcribed with oligo(dT) primer to produce cDNA [7], [30], [31], [32], [33], [35], [36], [37], [38], [39], [40], [41], [42], [44], [45], [46], [47], [48] (Figure 1A). The oligo(dT)-based methods are usually straightforward and easy to implement, and are therefore widely used for genome-wide APA studies. Moreover, some of these methods allow to multiplex large number of samples, if barcodes are introduced into library preparation, making them efficient for constructing numbers of libraries simultaneously [49].

Methods in this category differ from each other in several aspects, such as 5′ adapting, fragmentation, and second-strand synthesis. Wu et al. have developed a method to study APA in *Arabidopsis* [32]. Briefly, mRNA molecules are reversely transcribed with template switching, which introduces the cleavage sites of NlaIII and TaiI to the 3′ end of cDNA molecules. Second-strand synthesis is carried out via either PCR or Klenow DNA polymerase, and the resulting double-stranded DNA is digested with NlaIII and TaiI to generate smaller DNA fragments with sticky end. Such DNA fragments are then ligated with 5′ adaptor via those sites and amplified before sequencing. This method can detect small RNA molecules like miRNAs that are likely to be left out during size selection [35]. However, some RNA molecules that lack these restriction enzyme sites at their 3′ end would be missed by this method. Another widely-used method is poly(A) site sequencing (PAS-seq) [33]. To perform PAS-seq, poly(A)+ RNAs are fragmented, followed by reverse transcription with template switching to generate the first-strand cDNA molecules carrying 5′ and 3′ adaptors. After that, primers used in reverse transcription are removed, followed by cDNA amplification and size selection, and the resulting libraries are then ready for sequencing. The second method is easier to implement, and the random fragmentation of RNAs also avoids bias arising from enzymatic digestion. Besides, sequencing primers have already been introduced into the first-strand cDNA, which can serve as template for amplification without further ligation. Some other methods like poly(A)-seq and sequencing APA sites (SAPAS) are similar to PAS-seq in principle, with minor modifications in 5′ adapting, fragmentation, or sequencing primers [35], [37].

Other than priming at poly(A) tail of RNA molecules, oligo(dT) primers can also anneal to internal A-rich sequences, a phenomenon termed internal priming, leading to the generation of false pA peaks. Most of these fake pA peaks can be removed computationally based on the successive As in their downstream genomic sequences. However, such strategy would also lead to the systematical loss of real pA sites flanking A-rich sequences, which account for ∼ 8% of total pA sites in mouse transcriptome [4]. Recently, a method called whole transcriptome termini site sequencing (WTTS-seq) has been reported. Using PCR primers with additional Ts at the 3′ ends, the enriched cDNA molecules are reversely transcribed from poly(A)+ mRNA rather than from RNA molecules with internal A-rich sequences, thus reducing the effect of internal priming [42] (Figure 1A).

**Figure 2:**
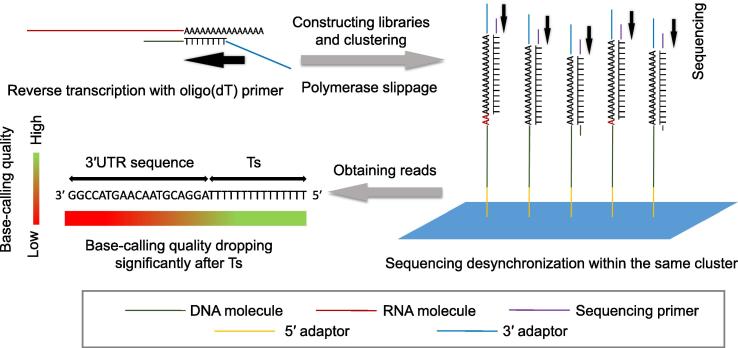
**Schematic diagram illustrating base-calling quality drop resulting from oligo(dT) primers** During library construction, T-stretch in oligo(dT) primers is transferred into final cDNA libraries. During library amplification or clustering, polymerase slippage might happen, giving rise to DNA molecules with different length of T/A-stretch. Therefore, sequencing desynchronization is likely to happen within the same cluster, resulting in significantly-decreased base-calling quality of nucleotides downstream of Ts. The figure was redrawn based on the principle of the method described previously [40]. Blue patch, Illumina flow cell surface.

The oligo(dT)-based methods have several shortcomings. Typically, when oligo(dT) primers are used in reverse transcription for 3′ end enrichment, the T-stretch of primers is retained during library construction, which has the benefit to preserve strand information, but can be problematic during sequencing. In Illumina sequencing platform, DNA molecules with homopolymers can probably lead to color imbalance and failure of cluster identification. Notably, base-calling quality significantly drops after sequencing through the T-stretch (Figure 2) [40]. This is likely due to the difference of sequencing starting point of molecules within the same cluster, a phenomenon called sequencing desynchronization (Figure 2) [40]. The drop of base-calling quality happens when sequencing starts from the termini corresponding to 3′ end of mRNA. Using fewer Ts in oligo(dT) primers can alleviate this defect; however, it will worsen the internal priming problem. Alternatively, libraries could also be sequenced from the termini corresponding to 5′ end of mRNA [39], [49], [50]. In this way, the base-calling quality won’t be influenced by T-stretch. Nonetheless, only a proportion of reads can reach pA sites, leading to shallower sequencing depth at poly(A) termini, which sometimes would cause the failure in calling some real pA sites.

**Figure 3:**
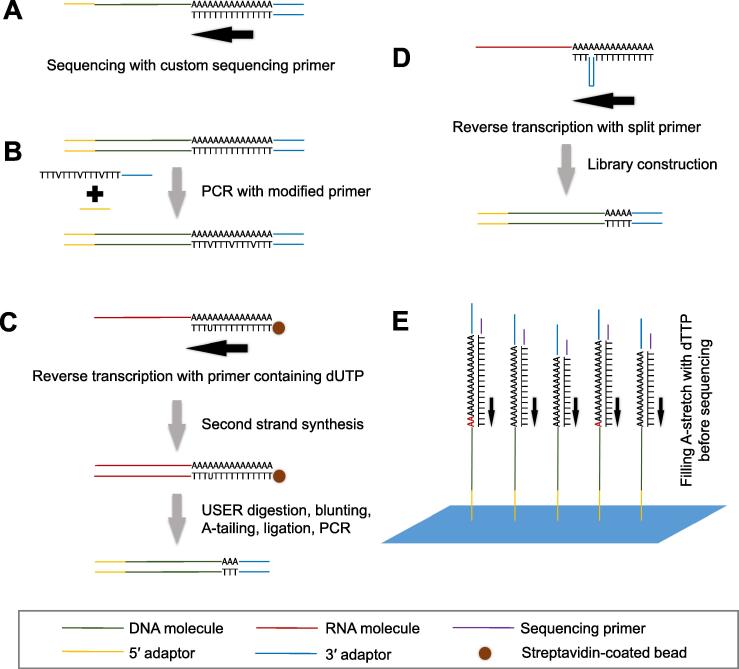
**Schematic diagram illustrating methods used to avoid sequencing directly through T-stretch** Methods using custom sequencing primers (**A**) or modified PCR primers (**B**) reduce the impact of T-stretch on sequencing. These methods cannot address the sequencing desynchronization problem. Methods using oligo(dT) primers containing dUTP (**C**) or a hairpin structure with 3′ adaptor sequences (**D**), or filling A-stretch with dTTP before sequencing (**E**) are able to avoid sequencing desynchronization. The figure is summarized based on the published methods mentioned in the main text. Blue patch, Illumina flow cell surface. USER, uracil-specific excision reagent.

Several approaches have been adopted to avoid sequencing directly through the T-stretch. Some methods used a custom sequencing primer, which contained several Ts at the 3′ end, to address this problem (Figure 3A) [33], [35], [50]. However, sequencing desynchronization could still occur because custom primer contains the fixed number of Ts, while the number of Ts in DNA molecules within clusters varies. Another widely-adopted approach uses a modified PCR primer during library construction, wherein several other nucleotides (A, C, or G) are introduced into the T-stretch in PCR primer (Figure 3B) [31], [37], which would keep nucleotides at similar proportion during sequencing and not affect clustering identification. However, this can alleviate but still could not solve the sequencing desynchronization problem completely. Several other methods have used modified reverse transcription primers to conquer the problem. In polyadenylation sequencing (PA-seq), oligo(dT) primers with a dTTP replaced with dUTP are used in reverse transcription, which are subsequently cleaved by uracil-specific excision reagent (USER) to remove most Ts (Figure 3C) [36], [38], [51]. Similarly, a method called A-seq uses a split primer, an oligo(dT) primer with a hairpin structure containing the sequence of 3′ adaptor inserted into a T-stretch, in reverse transcription (Figure 3D) [46]. Then PCR primers with 3′ adaptor sequences are used to amplify the libraries without T-stretch (Figure 3D). Besides, another genome-wide pA site mapping method termed 3′ T-fill, fills the A stretch of DNA molecules with unlabeled dTTPs just before sequencing, which allows sequencing directly from the pA sites, providing a simple method without using any modified or custom sequencing primers (Figure 3E) [40].

### RNA manipulation-based methods

RNA manipulation-based methods have been developed to circumvent internal priming caused by oligo(dT) priming. Typically, RNA fragments harboring pA site are enriched and adaptors are added to the 3′ end of these fragments. Then, primers annealing to the 3′ adaptors, rather than the oligo(dT) primers, are used for the reverse transcription (Figure 1B). In a method termed poly(A)-position profiling (3P-seq), complete mRNA is ligated with a splint adaptor and partially digested with RNase T1. Then, fragments with poly(A) tails are captured, and the poly(A) tails are reversely transcribed with dTTP only. Afterward, the poly(A) tails are digested with RNase H to release 3′-most RNA fragments without A-stretch. These fragments are subsequently ligated with 3′ and 5′ adaptors and prepared for Illumina sequencing (Figure 1B) [29]. 3P-seq is a well-designed method to solve the long-pending problem of internal priming, thus paving the way to identify genuine pA sites genome-widely. However, complex RNA manipulation steps are required, making this approach time-consuming and labor-intensive. Moreover, some studies have also reported that 3P-seq performs poorly in expression quantification [35].

3′ region extraction and deep sequencing (3′ READS) is another high-throughput method to discriminate poly(A) tails from internal A-rich regions using chimeric U5 and T45 (CU5T45) and a stringent primer washing condition [43]. This method solves the internal priming problem completely. Additionally, there comes a new method termed poly(A)-test RNA-sequencing (PAT-seq) lately. Different from the aforementioned methods focused on pA sites, PAT-seq relies on the poly(A) tail sequence of complete mRNA and is able to detect both the length of poly(A) tail and the location of pA site [30]. In PAT-seq, a 3′-tagging strategy is adopted to eliminate internal priming. Typically, total RNA is annealed to an oligo(dT) primer containing custom sequence at its 5′ end, then extended by Klenow polymerase using the primer as the template. Reverse transcription is then performed at higher temperature with the same primer. Oligo(dT) primers annealed to internal poly(A)-tracts are detached from RNA template due to low melting temperature, while those annealed to 3′-termini remain. PAT-seq has demonstrated its success in yeast [30]; however, further validation is needed for human RNA samples, which have an average poly(A) tail length of nearly 250 nucleotides [52].

There are currently no approaches apparently superior to the others. Each method has its own advantages and shortcomings. Although RNA manipulation-based methods circumvent the internal priming issue and identify true pA sites experimentally, Oligo(dT) priming-based methods can also eliminate most of internal fake pA sites computationally. There are always valuable findings regarding APA regulation by applying any of the methods described above. Detailed comparison of all aforementioned 3′-enriched RNA-seq methods is presented in Table 2. However, if one does need to know which the method of choice is, we would recommend those with most of the following features. First, random fragmentation rather than digestion using RNase or restriction enzymes is preferred for RNA fragmentation step, because some transcripts are insensitive to RNase or lack recognition sites of the restriction enzymes employed. Second, adaptor should be better introduced by reverse transcription, template switching, circularization, or DNA ligation, instead of RNA ligation, which could induce bias into libraries. Third, multiplexing sequencing is enabled during library construction, *i.e.*, library barcode is designed into primers or adaptors. Fourth, internal priming should be avoided or minimized, while 3′-most fragments are efficiently enriched. Fifth, sequencing should start from the end of pA site or from both ends. And last, the library construction protocol should be easy to implement and can be adapted for commonly-used sequencing platforms.

**Table 2:** **Details of each 3′-enriched RNA-seq method for global pA site profiling**

**Method**	**Fragmentation**	**Adapting**	**Sequencing desynchronization**	**Internal priming**	**Easy to implement**	**Sequencing platform**	**Ref.**
3SEQ	Heat shearing	DNA ligation	No	Yes	Medium	Illumina	[41]
Mangone et al.	DpnII	DNA ligation	Rare	Yes	Medium	454	[31]
3P-seq	–	RNA ligation	No	No	Low	Illumina	[29]
PAS-seq	Heat shearing	Reverse transcription with template switching	Yes	Yes	High	Illumina	[33]
SAPAS	Heat shearing	Reverse transcription with template switching	Rare	Yes	High	454/Illumina	[37]
Wu et al.	NlaIII or TaiI	DNA ligation	Yes	Yes	Medium	Illumina	[32]
MAPS	–	Reverse transcription and second strand synthesis	No	Yes	Medium	Illumina	[49]
Poly(A)-seq	–	Reverse transcription and second strand synthesis	Yes	Yes	High	Illumina	[35]
3′ seq	Heat shearing	DNA ligation	No	Yes	Medium	Illumina	[39]
A seq	RNase I	RNA ligation	No	Yes	Medium	Illumina	[46]
3′T fill	Heat shearing	DNA ligation	No	Yes	Medium	Illumina	[40]
3′ READS	Heat shearing	RNA ligation	Yes	No	Medium	Illumina	[43]
3PC	Heat shearing	Circularization	Yes	Yes	Medium	Illumina	[47]
PA-seq	Heat shearing	DNA ligation	No	Yes	Medium	Illumina	[36]
EXPRSS	Covaris shearing	DNA ligation	No	Yes	Medium	Illumina	[50]
PAT-seq	RNase T1	RNA ligation	Yes	No	Medium	Illumina	[30]
WTTS-seq	Heat shearing	Reverse transcription and second strand synthesis	Yes	Rare	Medium	Ion Torrent	[42]

The newly-published method WTTS-seq outperforms other oligo(dT) priming-based methods in terms of minimizing the effect of internal priming during library construction, and surpasses RNA manipulation-based methods by circumventing RNA ligation and complicated hands-on procedure. Moreover, this method has been optimized to increase transcriptome coverage and reduce sequencing bias, thus exhibiting high efficiency for global pA site analysis. However, the currently-used adaptors of WTTS-seq are matched to the Ion Torrent Personal Genome Machine, which is not so commonly-used as Illumina platform. Therefore, a modification enabling the WTTS-seq protocol to be compatible with Illumina HiSeq platform is highly wanted. On the whole, we recommend WTTS-seq for detecting authentic pA sites globally if it can be compatible with the Illumina platform.

### Bioinformatic analysis methods used in APA studies

After getting the read sequences that originate from the libraries for 3′ end of mRNA, a series of bioinformatics steps are needed to acquire the potential polyadenylation sites. First, the reads need to be aligned to the corresponding genome with or without the guide of annotated genes. Several tools can be used in this step, such as Bowtie [53], TopHat [54], STAR [55], and HISAT [56]. It should be noted that before aligning the reads back to genome, some filtration steps may be necessary to get rid of the noisy or adapter sequences introduced during the library preparation process. For paired-end reads, usually only the reads at one end are used for analysis in the next step, which are expected to originate from the 3′ end of an mRNA, depending on the strategies used during the library preparation and sequencing.

After mapping the right reads back to genome, the next step is to filter out reads that originate from internal priming as described above, which would finally lead to the false pA peaks. Two categories of methods are adopted to filter out internal-priming reads. The most widely-used one is based on the position of the aligned reads. A straightforward and idiomatic way is to filter out reads, alignment locations of which are just upstream of genomic A-rich sequences. For example, a consecutive 6As or 15As in 20 nucleotides are used in some studies [4], [36], [38]. However, it is worth noting that the exact threshold can be determined according to the real situation, no golden standard exists for now. Another method in the first category employs the Bayesian hypothesis model to infer the real pA sites and exclude the false ones, which also utilizes the ratio of adenosine in the genomic sequences downstream of the aligned reads [35]. The other type of methods used to filter out internal priming takes advantage of polyadenylation signals and their distribution in gene regions and the randomly-selected regions along the genome [57], [58]. However, some true pA sites that lack polyadenylation signals will be missed out when using this strategy. Therefore, to improve the filtering efficiency, a hybrid strategy is used in some studies by combining several published methods [31].

The next step is to predict the polyadenylation sites based on the aligned reads passing the filtration steps above. Peak-calling methods are usually adopted at this step. There are two strategies for peak calling, including window-based methods and density-based methods. Windows-based clustering methods were developed first, which count the number of reads within a window of certain length [4], [29], [33], [43]. On the other hand, density-based methods like F-seq generate a continuous tag density estimation to identify meaningful peaks, which can be trained to select an optimized window size and initiation position for peak calling [36], [37], [38], [59]. Methods of both categories have been used to identify meaningful pA sites in various studies.

After the acquisition of the possible pA sites, some methods employ statistical analyses, such as calculating the false-discovery rate (FDR) and polyA score, to evaluate the precision and sensitivity of peak-calling, to achieve a more precise identification of real pA sites [33], [35]. In addition, overlapping the identified pA sites obtained from each method with those in known polyA database is also a very useful and trustable evaluation method. With the identified pA sites at hand, pA site usages for each gene and their changes between samples can then be inferred. Different strategies have also been adopted at this step to get more intuitive quantification of pA site preference. These include the tandem 3′UTR isoform switch index (TSI) [37], [44], [60], effective 3′UTR length [36], [38], [60], and relative usage of distal pA site (RUD) [22], [60].

### 3′-enriched RNA-seq in single cell

Single-cell whole-transcriptome sequencing methods have been applied in many research fields, revealing extensive diversity in RNA expression between seemingly identical cells [61], [62]. Heterogeneity of gene expression among cells has been found to play an important role in many biological processes, such as embryonic development, immune cell activation, and cancer progression [63], [64], [65], [66], [67]. Different from bulk population RNA-seq, single cell RNA-seq (SCRS) uses a minute amount of total RNA from a single cell (typically 10 pg for a mammalian somatic cell, and can be as little as 0.5 pg for a T cell), which needs to be amplified to a sufficient amount for library construction. However, due to the minute quantity of the starting material in single-cell sequencing, amplification bias is unavoidable and worse than bulk population sequencing. To eliminate expression bias caused by whole transcriptome amplification and estimate the sensitivity of each SCRS method, unique molecular identifier (UMI) is integrated into primers during reverse transcription, serving as a molecular label of cDNA to filter out redundant DNA molecules that originate from overamplification [68].

Up till now, SCRS methods mainly focus on gene expression level, and few studies query post-transcriptional regulation mechanisms like APA, alternative transcription start site, and intron retention. It should be mentioned that to date, SCRS methods with the designed UMI are either 3′-enriched methods, such as CEL-seq2 [69], Drop-seq [70], automated massively parallel RNA single-cell sequencing (MARS-seq) [71] and single cell RNA barcoding and sequencing (SCRB-seq) [72], or 5′-enriched methods [68]. It is worth pointing out that these 3′-enriched methods used in SCRS, such as CEL-seq2 [69] and SCRB-seq [72], are also very suitable to be used for mining the pA site usage. However, the real capability of these methods in identifying pA sites needs to be evaluated in practice, and we are attempting to have a test that way. Besides, there are some widely-used methods without UMI incorporation, like Smart-seq2 [73] and commercial SMART-seq v4 protocol (Clontech). These methods are able to sequence whole transcripts but have limited capacity in detecting pA sites.

Recently, a new method termed BATSeq, which combines a conventional SCRS protocol and a bulk population 3′-enriched RNA-seq protocol, has been developed to survey the genome-wide polyadenylation in single cells [74]. Briefly, poly(A)+ RNA of single cells is amplified to microgram level via a modified Quartz-seq method, a SCRS method with high reproducibility, high efficiency, and few PCR byproducts [75]. The amplified RNA then serves as the starting material for library construction via the established protocol with modifications [76]. Using BATSeq, the authors reveal a heterogeneity of pA site usage among single cells. However, due to its low sensitivity (approximately 5%), BATSeq has to utilize a very complicated analysis pipeline to discover real biological variation among single cells [74]. Moreover, due to the multiple steps of amplification, batch effects, and long hands-on time featured by Quartz-seq, it is hard for BATSeq to be quantitative even when external spike-ins are added. We hope that more promising progress on APA study at the single-cell level will come up in the near future.

## Functions of APA

**Figure 4:**
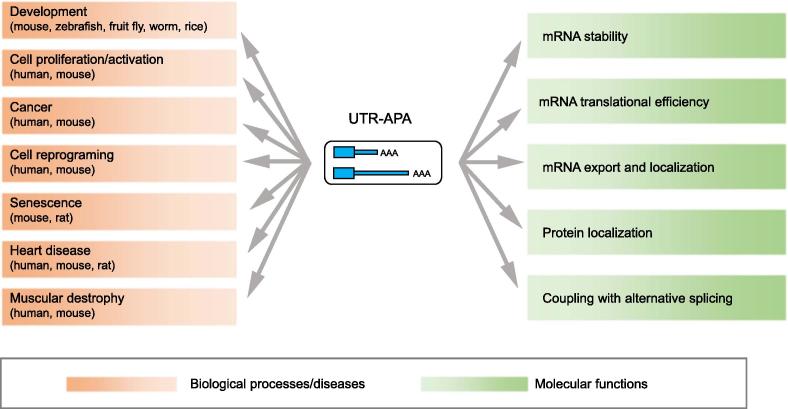
**Schematic diagram illustrating functional consequences of UTR-APA** The physiological/pathological processes (on the left) and molecular functions (on the right) of UTR-APA reported so far are illustrated in the figure. APA has been found to play a role in diverse biological processes, summarized as development [17], [31], [44], [83], [100], [101], [102], cell proliferation or activation [14], [105], [106], cell reprogramming [16], cellular senescence [60], *etc*. Besides, APA has also been reported to occur in various pathological processes, such as cancer [23], [25], [107], [108] heart diseases [109], [110], muscular dystrophy [39], [89] and amyotrophic lateral sclerosis. With regard to molecular functions, APA regulates gene expression by affecting stability, translational efficiency, export, and localization of mRNA, as well as protein localization, and by functionally coupling with alternative splicing. APA, alternative polyadenylation.

As a post-transcriptional event, APA has important roles in gene expression regulation. APA has been reported to affect the expression of genes containing multiple pA sites, through impacting mRNA metabolism (*e.g.*, degradation rate, translation efficiency, export, and localization) and protein localization (Figure 4) [2], [3], [7], [8], [9]. The molecular consequences of APA are further manifested as cellular phenotypes such as cell proliferation rate [14] and cell identity [24], [36]. Since APA can be divided into two main types (3′UTR and non-3′UTR) based on their genomic location, the functions of APA are then discussed accordingly as bellow (Figure 4).

### APA in 3′UTR

Alternative pA sites in mouse and human genome have been shown to be located mainly in 3′UTRs (UTR-APA) [35], which often harbor various regulatory sequences. This type of APA can change the length and composition of 3′UTR and impact the binding of certain miRNAs or RBPs, leading to difference in mRNA stability, export, localization, translational efficiency, *etc*. Moreover, a recent report has demonstrated that UTR-APA can also affect protein localization, providing a novel function of APA [7]. The gene encoding membrane protein CD47 has alternative 3′UTR. CD47 derived from longer isoform is highly expressed in the cell membrane, while CD47 generated from the shorter isoform is primarily localized in endoplasmic reticulum [7].

Notably, although genes with longer 3′UTR tend to show a decreased steady-state expression level under certain condition [77], it does not necessarily mean that every single gene with longer 3′UTR is less stable than those with the shorter one. A cell-cycle gene *polo*, which encodes polo-like kinase in *Drosophila*, is such an example. The longer 3′UTR isoform of *polo* generates more protein than the shorter one [78]. miRNAs targeting 3′UTR are known to either cause mRNA unstable or reduce the translation efficiency. However, the lower degradation rate and higher protein production of *polo* gene with longer 3′UTR can’t be explained by the interaction with miRNAs. Previous studies have reported that RBPs or RNA secondary structure may be responsible for upregulation of many genes [79]. Their potential roles in regulating *polo* gene expression deserve further studies.

### APA in non-3′UTR

An increasing number of studies have reported that some alternative pA sites occur in genomic regions other than annotated pA sites and 3′UTRs in many eukaryotic organisms [3], [4]. These include 5′UTRs, introns, and coding sequences (CR-APA), as well as intergenic regions, offering new insights into the underlying mechanisms for non-3′UTR polyadenylation and its regulatory roles. Among them, intronic pA sites attract increasing interest. Mueller et al. have recently discovered that intronic polyadenylation of the gene encoding platelet-derived growth factor receptor α (*Pdgfra*) in fibro/adipogenic progenitors causes the elevated expression of a shorter transcript variant with a truncated kinase domain, which reduces tissue fibrosis through regulating pro-fibrotic pathways in muscle [80]. UV treatment of mammalian cells induces a significant activation of intronic polyadenylation in genes relevant to DNA damage response (DDR) including RNA polymerase II (DNA directed) polypeptide A (*POLR2A*), cyclin-dependent kinase inhibitor 1A (*CDKN1A*), and ephrin B2 (*EFNB2*). Such increased usage of intronic pA sites is caused by a decrease in U1 small nuclear ribonucleoprotein (U1 snRNP) levels in UV-induced DDR [81]. It is worth noting that U1 snRNP is the first protein reported to protect the premature termination of mRNA by cryptic intronic pA sites [82]. In addition, by performing APA study during mouse retinal development, we and our collaborators have revealed 3′UTR lengthening and increased usage of intronic pA sites over the process of retinal development [83]. Notably, we have also identified a considerable number of polyadenylated long ncRNAs (lncRNAs) co-expressed with protein-coding genes that are involved in retinal cell development [83]. In line with this study, similar percentage of APA events in both mRNAs and lncRNAs has also been reported in other studies [36], [43]. The exact functions of APA in lncRNAs deserve further investigation.

## The relationship between APA and AS

One line of compelling evidence indicating crosstalk between APA and AS under certain circumstance is that some factors involved in AS like snRNP proteins are reported to interact with *trans*-acting factors associated with APA. For example, in human cells, U1 snRNP interacts with cleavage factor I (CF Im) [84], U2 snRNP interacts with cleavage and polyadenylation specificity factor (CPSF) [85], and the 65 kDa subunit of U2 snRNP auxiliary factor (U2AF65) associates with CF Im [86]. Besides snRNP proteins, serine/arginine rich proteins (SR proteins) are other AS regulating factors [87], which have been reported to function in APA regulation recently [88]. Interestingly, serine and arginine rich splicing factor 3 (SRSF3) and SRSF7 play an opposite role in regulating the length of 3′UTR of 32 target genes. Down-regulation of SRSF3 leads to shortening of 3′UTR, while depletion of SRSF7 leads to lengthening of 3′UTR [89]. Another example of this crosstalk is heterogeneous nuclear ribonucleoprotein A2/B1 (hnRNPA2B1), which regulates both APA and AS in spinal cord [90]. HnRNPA2B1 interacts with UAGG motifs in 3′UTRs to affect APA. Also, depletion of hnRNPA2B1 results in removal of the intron of the gene encoding arginine/serine-rich protein 1 (*Rsrp1*) [90].

Other than endogenous proteins, some peptides from viruses can also be responsible for interaction between APA and AS. For example, herpes simplex virus (HSV) infected cell polypeptide 27 (ICP27) promotes the 3′ end formation of co-transcriptional pre-mRNA using cryptic polyadenylation signals in introns, thus generating hundreds of novel, intron-less, GC-rich cellular transcripts that resemble HSV genes [91]. ICP27 also causes aberrant pre-mRNA splicing of some host genes, suggesting an overlapping mechanism for ICP27-mediated aberrant pre-mRNA splicing and polyadenylation [91].

In general, splicing factors promote the usage of UTR-APA but suppress the cleavage of intronic pA sites [92]. Tian et al. have firstly revealed that around 20% human genes contain intronic pA sites [93], suggesting that negative interaction between splicing factors and intronic pA sites might be very common. In fact, multiple studies have shown that inhibition of polyadenylation by splicing factors like U1 snRNP is coupled with the up-regulation of transcripts bearing intronic pA site [94], [95], [96], [97]. Another study has illustrated that splicing factor hnRNP H suppresses cryptic pA site selection but activates distal 3′ splicing site to generate the specific isoform of the gene encoding acetylcholinesterase (*ACHE*), probably by competing with the 3′ end processing factor cleavage stimulation factor 64 kDa (CstF64) [98]. In addition to *ACHE*, similar phenomena have also been observed in other genes as well [98].

Recently, a full-length cDNA sequencing method termed ISO-seq has been established by Pacific Bioscience, allowing the direct sequencing of full-length transcripts without the need for assembly [99]. ISO-seq shows unique scientific value and will open new exciting research area, such as interrogating the association between AS and APA, which is still difficult to dissect using NGS platforms only. It is expected that with the advances in deep sequencing technologies, more extensive and in-depth findings related to APA and AS will be coming.

### APA in biological and pathological processes

Global APA changes have been discovered in numerous physiological processes including development, cell differentiation [17], [31], [44], [83], [100], [101], [102], cell/tissue identity [35], [36], [103], [104], cell proliferation [14], [105], [106], neuron activation [15], cell reprogramming [16], and cell senescence [60], as well as in pathological processes including cancer [23], [37], [107], [108], viral infection [38], cardiac hypertrophy [109], heart failure [110], oculopharyngeal muscular dystrophy [39], and amyotrophic lateral sclerosis [90] (Figure 4). For all these physiological or pathological processes, APA in 3′UTR takes the most part and has been intensively studied than that in non-3′UTR regions. Since APA in 3′UTR leads to the generation of mRNAs with different length of 3′UTR, we focused on global alteration of APA in 3′UTRs as below (Table 3).

**Table 3:** **Global alteration of 3′UTR length regulated by APA in biological processes and diseases**

**Alteration of 3′UTR length**	**Processes**
Global shortening of 3′UTRs	Cell proliferation; Neuron activation; Tumorigenesis; Cardiac hypertrophy; Oculopharyngeal muscular dystrophy

Global lengthening of 3′UTRs	Mouse embryonic development and myogenesis; Drosophila embryogenesis; Cellular senescence

Dynamic changes of 3′UTR length	Cell/tissue identity; Zebrafish embryogenesis; Mouse retinal development; Cell reprogramming; Heart failure

### Global shortening of 3′UTRs

Studies have shown that genome-wide preference of promoter-proximal pA sites leads to global shortening of mRNA 3′UTRs, which occurs in a series of biological processes, especially in cancer. For example, Mayr and Bartel have revealed that a considerable number of oncogenes in cancer cells exhibit 3′UTR shortening phenomenon. The isoform of certain oncogenes with shorter 3′UTR, such as *cyclinD1* (*CCND1*), *Dicer1*, and the gene encoding insulin-like growth factor 2 mRNA binding protein 1 (*IGF2BP1/IMP-1*), show increased stability and produce more proteins [108]. Furthermore, 3′UTR shortening-mediated upregulation of *IGF2BP1/IMP-1* could lead to cellular phenotypes such as oncogenic transformation, demonstrating the importance of APA regulation in cancer [108]. Bioinformatics analysis on regular RNA-seq data reveals that as high as 91% genes with APA containing shorter 3′UTRs are found in cancer [23]. Most importantly, CstF64, a polyadenylation factor bound downstream of pA sites, is discovered to be a potential regulator for 3′UTR shortening across multiple cancer types [23]. Another study also confirms the phenomenon that cancer tissues exhibit a global shortening at 3′UTR in comparison with the matched normal tissues. Genes with shorter 3′UTR and upregulated expression in cancers are enriched in cell–cell and/or cell-ECM (extra-cellular matrix) pathways [47]. However, there also exist cancer cell lines that do not exhibit global 3′UTR shortening. For example, switching of different pA sites is observed between two human breast cancer cell lines, MCF7 and MB231. Genes tend to be short in MCF7 but tend to be long in MB231 [111]. In addition, global 3′UTR shortening also occurs in other biological processes as well, such as T cell activation [14] and cardiac hypertrophy [109].

DNA mutations are usually considered as the cause of tumors, and have been recently discovered to impact APA switch in cancer. For example, in the *CCND1* locus, a point mutation downstream of stop codon creates a novel polyadenylation site and therefore leads to an *CCND1* isoform with a shorter 3′UTR [112]. This mutation correlates with increased cyclin D1 expression and poor survival rate of patients with mantle cell lymphoma [88]. A recent genome-wide association study (GWAS) analysis highlights the importance of polyadenylation in tumor development and progression. It is found that the most significant single nucleotide polymorphism (SNP) associated with basal cell carcinoma is located in the 3′UTR region of the oncogene encoding the well-known tumor protein P53 (*TP53*), which converts the canonical pA signal ‘AATAAA’ into ‘AATACA’, inducing the damaged 3′-end formation of *TP53* mRNA. This SNP is further identified to be associated with other tumors, such as prostate cancer, malignant glioma, and colorectal adenoma, but not breast cancer [113].

### Global lengthening of 3′UTRs

Conversely, genes favoring promoter-distal pA sites would generate transcripts with 3′UTR lengthening globally. Progressive lengthening of 3′UTRs was first discovered during the embryonic development process [17] and was recapitulated in C2C12 myoblast cells [17]. Further evidence suggests that reduced polyadenylation of mRNA is likely the cause of such 3′UTR lengthening [17]. Hilgers et al. have discovered that a subset of neural-specific genes undergo 3′UTR elongation during *Drosophila* embryogenesis. Moreover, some extended 3′UTRs contain potential RBP recognition motifs that act as translational repressors, such as Pumilio regulatory element [101]. Besides, we have also detected progressive 3′UTR lengthening of mRNAs during cellular senescence in mouse and rat cells, accompanied by a decreased expression of polyadenylation factors. Intriguingly, genes undergoing 3′UTR lengthening share common signaling pathways related to cell senescence [60].

### Dynamic changes of 3′UTRs length

Despite the unidirectional trend of APA change described above, dynamic changes of 3′UTR length can also occur, especially during a long-period process with multiple time points. Zebrafish development is one of such examples. During the zygotic genome activation in early embryogenesis of zebrafish, a global shortening of 3′UTRs is observed, while a quick lengthening of mRNA 3′UTRs appears during the following stage of gastrulation [44]. A temporal regulation model coordinated by APA and *trans-*acting factors has thus been proposed. Dynamically-regulated APA is also found during mouse retinal development and maturation process [83]. The reprogramming of different cell types into induced pluripotent stem cells (iPSCs) is also accompanied by either global lengthening (spermatogonial cells) or shortening (remaining somatic cells) of 3′UTRs [16]. However, apparent overall shortening/lengthening patterns of APA have not been seen in some specimens. For example, among the 1370 genes with APA changes in the failing human heart, about one half favor distal pA site and the other half prefers proximal pA site [110].

Altogether, these aforementioned findings clearly demonstrate that APA changes can accompany many biological processes and probably play important roles. With more extensive studies on various samples, more featured pA sites and functional pathways involved will be discovered, and the biological significance of APA in many developmental or pathological processes would gradually heave in sight as well.

## Concluding remarks

Alternative polyadenylation, alternative promoter and alternative splicing, are the three major types for gene expression regulation at RNA level, covering the starting to the ending of transcription, which contribute to the diversity and complexity of transcriptome and proteome. Recent years have witnessed the spring up of APA studies and related interesting findings. For example, more than 70% of the human genes have multiple pA sites, suggesting the prevalence of APA across the genome. APA has also been found to accompany many biological processes and can play important roles under given conditions. Currently quite a few NGS-based methods have been developed for detecting pA sites genome-widely, with each having advantages and shortcomings of its own. More accurate and practical methods are still desired, such as the methods that can circumvent internal priming and that have the ability to identify real pA site precisely, and that are practical for study in single cells. APA has demonstrated its universal and important roles both at molecular function level and physiological level. Global APA regulation has been found involved in the increasing number of biological and pathological processes. However, omics studies need to be combined with candidate gene strategy to reveal the underlying biological function and detailed regulation mechanisms for given genes and conditions. For example, isoforms with longer 3′UTR for a considerable number of genes generate more protein than those with shorter 3′UTR. Do RBPs play a role in explaining this phenomenon? And if so, which RBP and how it functions to regulate? These concerns remain to be elucidated. With more researchers diving into this field, more functions and biological roles, along with in-depth regulatory mechanisms of APA, will be discovered.

## Competing interests

The authors declared that there are no competing interests.
